# Bioactive Properties of Selected European *Phellinus* Species: A Comprehensive Study

**DOI:** 10.3390/ijms26168013

**Published:** 2025-08-19

**Authors:** Grzegorz Świderski, Monika Kalinowska, Ewa Zapora, Marek Wołkowycki, Marcin Stocki, Ewa Ciszkowicz, Aleksandra Bocian, Marcin Jaromin, Mirosław Tyrka, Katarzyna Lecka-Szlachta, Elżbieta Wołejko, Urszula Wydro, Małgorzata Pawłowska, Paweł Golianek, Małgorzata Zawadzka, Qëndrim Ramshaj, Carolina Elena Girometta, Mitko Karadelev

**Affiliations:** 1 Department of Chemistry, Biology and Biotechnology, Bialystok University of Technology, Wiejska 45E, 15351 Białystok, Poland; m.kalinowska@pb.edu.pl (M.K.); e.wolejko@pb.edu.pl (E.W.); u.wydro@pb.edu.pl (U.W.); malgorzata.zawadzka@pb.edu.pl (M.Z.); 2 Department of Silviculture and Forest Utilization, Bialystok University of Technology, Wiejska 45E, 15351 Bialystok, Poland; e.zapora@pb.edu.pl (E.Z.); m.wolkowycki@pb.edu.pl (M.W.); m.stocki@pb.edu.pl (M.S.); 3 Department of Biotechnology and Bioinformatics, Faculty of Chemistry, Rzeszow University of Technology, Powstańców Warszawy 6, 35959 Rzeszow, Poland; eciszkow@prz.edu.pl (E.C.); bocian@prz.edu.pl (A.B.); mjaromin@prz.edu.pl (M.J.); mtyrka@prz.edu.pl (M.T.); szlachta@prz.edu.pl (K.L.-S.); 4 Faculty of Environmental Engineering, Lublin University of Technology, Nadbystrzycka 40B, 20618 Lublin, Poland; m.pawlowska@pollub.pl (M.P.); p.golianek@pollub.pl (P.G.); 5 Department of Biology, Faculty of Mathematics and Natural Sciences, University of Prishtina, St. “Eqrem Çabej”, 10 000 Prishtina, Kosovo; qendrim.ramshaj@uni-pr.edu; 6 Department of Earth and Environmental Sciences, University of Pavia, Via Sant’Epifanio 14, 27100 Pavia, Italy; carolinaelena.girometta@unipv.it; 7 Institute of Biology, Faculty of Natural Sciences and Mathematics, St. Cyril and Methodius University, Arhimedova 3, 1000 Skopje, North Macedonia; mitkok@pmf.ukim.mk

**Keywords:** *Phellinus*, medical fungi, cytotoxic study, antimicrobial study

## Abstract

This study conducted a multi-directional evaluation of the chemical potential and biological properties of selected European fungal species of the genus *Phellinus*. We investigated 30 samples belonging to 22 *Phellinus* species. Fruiting bodies were collected, among other specimens, in the Białowieża Forest (Poland); Village Kozhle (North Macedonia); Estremadura, Sesimbra, and Lagoa de Albufeira (Portugal); Zlatari close to Prishtina (Kosovo); and Spoleto and the Bosco Siro Negri State Nature Reserve (Italy). Morphological identification of the collected fungi was carried out, and genetic tests were performed to confirm the identity of the collected specimens. Methanol extracts for biological activity tests were prepared. Screening of antimicrobial activity of 30 methanolic extracts was performed on strains of bacteria (*Klebsiella pneumoniae*, *Pseudomonas aeruginosa*, *Staphylococcus aureus*, *Staphylococcus epidermidis*, and *Kocuria rhizophila*) and fungi (*Candida albicans*). Antioxidant activity tests (DPPH and ABTS) were also performed. The three most biologically active fungi species were selected (*Phellinus igniarius*, *Fomitiporia robusta*, and *Porodaedalea pini*) for further research. The chemical composition of the extracts was determined using GC-MS analysis. Thermal decomposition studies and spectroscopic analysis of the dry fruiting bodies were performed. The extracts were tested for their antimicrobial activity against antibiotic-resistant bacteria. Cytotoxic activity was also tested.

## 1. Introduction

The genus *Phellinus sensu lato* is a significant group within the family Hymenochaetaceae Imazeki & Toki, comprising approximately 220 species globally [1,2]. These fungi are predominantly lignicolous, meaning they grow on wood, and are known for their roles in forest ecosystems as both decomposers and pathogens [3]. *Phellinus* species are found in diverse habitats, including temperate and tropical forests, where they contribute to the decomposition of wood and nutrient cycling [4]. In Europe, several species of *Phellinus* are recognized for their ecological and medicinal importance, with some species having been used in traditional medicine for centuries [5].

Taxonomically, *Phellinus sensu lato* is a complex genus with a rich diversity of species, characterized by their perennial fruiting bodies and tough, woody consistency [4]. The genus has been subject to extensive taxonomic revisions, with recent studies employing molecular phylogenetic analyses to better understand the relationships within the group [6]. These analyses have led to the reclassification of some species and the identification of new ones, highlighting the genus’s diversity and evolutionary history [7].

### 1.1. Chemical Composition

The genus *Phellinus*, a group of medicinal fungi, is known for its diverse chemical composition—particularly its secondary metabolites. These fungi produce a wide array of compounds, including polysaccharides, flavones, coumarins, terpenes, steroids, and styrylpyranones, which have been extensively studied for their potential health benefits [1]. Among these, polysaccharides are the most prominent bioactive macromolecules, exhibiting various health-promoting effects such as immunomodulatory, anticancer, and antioxidant activities [8,9]. Specific species such as *Phellinus igniarius* (L.) Quél., *Porodaedalea pini* (Brot.) Murrill [former *Phellinus pini* (Brot.) A. Ames], *Phellinus tuberculosus* (Baumg.) Niemelä [syn. *P. pomaceus* (Pers.) Maire], and *Fomitiporia robusta* (P. Karst.) Fiasson & Niemelä [former *P. robustus* (P. Karst.) Bourdot & Galzin] have been found to contain polysaccharides, phenolic compounds, and terpenoids that contribute to their biological activities, including anticancer and antiviral effects [5]. *Sanghuangporus baumii* (Pilát) L.W. Zhou & Y.C. Dai [former *Phellinus baumii* Pilát] has yielded unique sesquiterpenes and alkyl-benzene derivatives, such as phellinbaumins A and B, which have shown moderate anti-inflammatory activity [10]. *Fuscoporia gilva* (Schwein.) T. Wagner & M. Fisch. [former *Phellinus gilvus* (Schwein.) Pat.] contains significant amounts of carbohydrates, proteins, and phenols, which contribute to its antioxidant potential [11]. *Tropicoporus linteus* (Berk. & M.A. Curtis) L.W. Zhou & Y.C. Dai [former *Tropicoporus linteus* (Berk. & M.A. Curtis) Teng] is notable for its acidic proteo-heteroglycan—a complex molecule composed of polysaccharides and proteins—which has been isolated and characterized for its unique structural properties [12]. Additionally, *P. igniarius* has been found to produce pyrano [4,3-c] isochromen-4-one derivatives, such as phelligridins H, I, and J, which possess novel carbon skeletons and exhibit cytotoxic activity against cancer cell lines [13]. These findings highlight the chemical diversity within the genus *Phellinus* and underscore the potential of these fungi as sources of novel bioactive compounds.

### 1.2. Bioactivity

**Anticancer activity**

*Phellinus sensu lato* species, particularly *Tropicoporus linteus* and *Phellinus nigricans*, (Fr.) P. Karst. (the latter being currently embedded into *P. igniarius* according to both Mycobank and Index Fungorum) have been extensively studied for their anticancer properties, with several in vivo studies highlighting their potential. In an in vivo study, polysaccharides extracted from *PT. linteus* significantly reduced tumor growth in a mouse model by modulating the Wnt/β-catenin signaling pathway, which is crucial for cell proliferation and survival [14]. Additionally, *P. linteus* extracts have been found to selectively induce apoptosis in cancer cells while exhibiting low toxicity towards normal cells, suggesting a potential for targeted cancer therapy [15]. Proteoglycans isolated from the mycelium of *P. nigricans* have demonstrated antitumor effects in vivo against Sarcoma 180 in mice. These effects were primarily attributed to the immunomodulatory properties of the proteoglycans, which enhanced the immune response rather than directly killing the tumor cells. The study by the authors of [16] reported increased spleen and thymus weights and elevated levels of tumor necrosis factor-alpha (TNF-α), indicating an activated immune system capable of combating tumor growth. Moreover, hispolon—a compound found in *Tropicoporus linteus*—has been identified as a potent anticancer agent. In vivo studies have shown that hispolon can induce apoptosis in cancer cells by disrupting mitochondrial membrane potential and activating caspase pathways, which are essential for programmed cell death [17]. This compound has also been isolated from other *Phellinus* species, including *P. igniarius*, *Sanghuangporus. lonicerinus*, (Bondartsev) Sheng H. Wu, L.W. Zhou & Y.C. Dai [fromer *P. lonicerinus* (Bondartsev) Bondartsev & Singer], *Fulvifomes. merrillii,* (Murrill) Baltazar & Gibertoni [former *P. merrillii* (Murrill) Ryvarden], and *Inonotus hispidus*,(Bull.) P. Karst. and has been shown to modulate several signaling pathways involved in cancer progression, such as cell cycle arrest and inhibition of angiogenesis and metastasis [18].

**Immunomodulatory activity**

Selected *Phellinus* species also demonstrate notable immunomodulatory effects. Polysaccharides have been shown to enhance both hormonal and cell-mediated immune functions, which can help in managing various immune-related conditions [2,9,19], *Tropicoporus linteus* polysaccharides have demonstrated significant anti-inflammatory effects by inhibiting pathways such as MAPK and NF-kappaB, which are involved in the expression of inflammatory cytokines. This inhibition helps to reduce inflammation in various models, including those induced by lipopolysaccharides (LPSs) [20,21,22,23]. Additionally, *Tropicoporus linteus* has been shown to modulate immune responses by increasing the production of cytokines such as IL-2, IL-12, and IFN-gamma, which are crucial for immune function [20,21,22,23]. *Sanghuangporus baumii* also exhibits anti-inflammatory properties, particularly in conditions such as inflammatory bowel disease. Its polysaccharides can decrease the expression of pro-inflammatory genes and increase anti-inflammatory markers, contributing to tissue repair and immune modulation [24,25]. This species has been noted for its potential in enhancing immune responses, especially in immunosuppressed conditions, by improving lymphocyte proliferation and natural killer cell activity [24].

**Antioxidant activity**

The genus Phellinus is known for its diverse species that exhibit significant antioxidant activities, which have been the focus of various studies. *Porodaedalea pini* and *Phellinus sp*. collected in Foshan have demonstrated potent antioxidant activities, with IC50 values for DPPH and hydroxyl radicals comparable to ascorbic acid, indicating their strong radical scavenging capabilities [26]. Similarly, *Fuscoporia torulosa* (Pers.) T. Wagner & M. Fisch. [former *Phellinus torulosus* (Pers.) Bourdot & Galzin] and *P. igniarius* have been used to fortify yogurt, enhancing its antioxidant profile significantly, with *P. torulosus* showing superior DPPH inhibition and lipid peroxidation neutralizing activities [27]. *Sanghuangporus baumii* extracts, which are rich in phenolics, have shown high antioxidant and free radical scavenging activities, particularly in methanol and hot water extracts, exhibiting 80–90% inhibition rates in various assays [28]. The ethanolic extracts of *S. baumii* have been effective in protecting PC12 cells from oxidative damage, with strong correlations between flavonoid content and antioxidant capacity [29]. *Phellinus rimosu*s (Berk.) Pilát has also been noted for its significant antioxidant and antihepatotoxic activities, with its ethyl acetate extract showing potent free radical scavenging and liver protective effects [30]. *Tropicoporus linteus*, extracted with ethanol, has demonstrated strong antioxidant activities, comparable to vitamin C, and has shown potential antitumor effects due to its anti-angiogenic properties [31]. In studies conducted in Northeast Thailand, *Fuscoporia torulosa* and *Porodaedalea pini* exhibited potent radical scavenging activities, with high total phenolic content correlating with their antioxidant efficacy [32]. Additionally, *Phellinus rimosus* has shown excellent antioxidant and antimutagenic activities, particularly in ethanol extracts, suggesting its potential in disease prevention and treatment [33].

**Antiviral activity**

Among the species studied, *T. linteu*s and *P. igniarius* have shown promising antiviral effects. *Tropicoporus linteus*, for instance, has been investigated for its potential against dengue virus type-2, where compounds such as phelignan A and dimeric ferulates exhibited significant inhibitory activity [34]. Additionally, *Tropicoporus linteus* has demonstrated potential in inhibiting SARS-CoV-2, with key metabolites such as hispidin and hypholomine B reducing viral entry by affecting angiotensin-converting enzyme 2 gene expression [35]. *Phellinus igniarius* has also been highlighted for its antiviral properties. A new sesquiterpenoid isolated from this species showed significant activity against the H5N1 influenza virus, suggesting its potential as a neuraminidase inhibitor [14]. Furthermore, polysaccharides and flavonoids derived from *Phellinus igniarius* have been shown to enhance immune responses in immunocompromised mice, which could indirectly contribute to antiviral defense [19].

**Antidiabetic effects**

*Phellinus* spp. have shown promising antidiabetic effects in various in vivo studies. *Tropicoporus linteus* polysaccharides (PLPs) have been demonstrated to ameliorate insulin resistance in mice fed a high-fat, high-fructose diet. The administration of PLP significantly reduced fasting blood glucose levels and improved glucose intolerance. This effect was attributed to the modification of hepatic phospholipid metabolism and the enhancement of insulin signaling transduction, as well as the stimulation of gut bacteria capable of synthesizing vitamin B12, which plays a role in metabolic processes [36]. Similarly, *S. baumii* exopolysaccharides (EPSs) have exhibited substantial hypoglycemic effects in streptozotocin-induced diabetic rats. The EPS treatment led to a significant reduction in plasma glucose levels and improved liver function, as indicated by decreased activities of liver enzymes such as alanine aminotransferase and aspartate aminotransferase. These findings suggest that *S. baumii* EPS could be beneficial for managing diabetes mellitus [37]. *Phellinus igniarius* has also been studied for its antidiabetic properties. A polyphenol-rich extract from this species was shown to lower fasting blood glucose levels and improve glucose tolerance in KK-Ay mice with spontaneous type 2 diabetes. The extract enhanced the expression of GLUT4 and activated the AMPK pathway; these processes are crucial for glucose uptake and metabolism [38]. Furthermore, *S. baumii* extract (PBE) demonstrated hypoglycemic effects in diabetic mice by reducing fasting blood glucose levels and improving insulin sensitivity. The extract modulated the gut microbiota, promoting beneficial bacteria and inhibiting harmful ones, which may contribute to its antidiabetic effects [39].

*Phellinus* species are valuable medicinal fungi with a wide array of health-promoting properties, including antitumor, immunomodulatory, anti-inflammatory, antioxidant, and antidiabetic effects. These properties are primarily attributed to their rich composition of bioactive compounds, making them a significant focus of research for developing functional foods and pharmaceuticals.

The aim of this study was a multi-directional evaluation of the chemical potential and biological properties of selected European fungal species of the genus *Phellinus*.

As part of the project, 30 specimens of *Phellinus* mushrooms were collected. Morphological identification of the collected fungi was carried out, and genetic tests were performed to confirm the identity of the collected specimens. Methanol extracts for biological activity tests were prepared. Screening of the antimicrobial activity of 30 methanolic extracts was performed on strains of bacteria (*Klebsiella pneumoniae*, *Pseudomonas aeruginosa*, *Staphylococcus aureus*, *Staphylococcus epidermidis*, and *Kocuria rhizophila*) and fungi (*Candida albicans*). Antioxidant activity tests (DPPH and ABTS) were also performed.

Three species of fungi with the highest activity were selected for further research (*Phellinus igniarius*, *Fomitiporia robusta*, and *Porodaedalea pini*) The chemical composition of the extracts was determined using GC-MS analysis. Thermal decomposition studies and spectroscopic analysis of the dry fruiting bodies were performed. The extracts were tested for their antimicrobial activity against antibiotic-resistant bacteria. Cytotoxic activity was also tested.

## 2. Results and Discussion

### 2.1. Antimicrobial Activity—Preliminary Studies

The antimicrobial activity of 30 samples of methanolic extracts of fruiting bodies of the genus *Phellinus* was tested. The antimicrobial properties of the tested fungal extracts are summarized in Table 1. Based on the obtained results, it was observed that the highest antimicrobial activity was shown by *Fomitiporia robusta* (mainly on strains *C. albicans*—MIC = 25 mg/mL, *S. aureus*—MIC = 25 mg/mL, and *K. rhizophila*—MIC = 25 mg/mL); in turn, *Porodaedalea pini* inhibited the growth of strains *C. albicans* (MIC = 12.5 mg/mL), *S. aureus* (MIC = 25 mg/mL), *K. rhizophila* (MIC = 25 mg/mL), and *S. epidermidis* (MIC = 25 mg/mL). Extract from *Phellinus igniarius* was the most effective against *S. aureus* (MIC = 6.3 mg/mL), *S. epidermidis* (MIC = 25 mg/mL), and *C. albicans* (MIC = 25 mg/mL).

### 2.2. Elemental Analysis

The highest amounts of macroelements (P, K, and Ca) were found in *Fomitiporia robusta* extract (2930.88 mg/kg, 3218.03 mg/kg, and 324.89 mg/kg of extract weight) (Table 2). The potassium content in the extracts from *P. pini* and *P. igniarius* was at a similar level, but was significantly lower than in the extract from *F. robusta*. The lowest phosphorus content was recorded in the extract from *P. pini* (1224.77 mg/kg); in the extract from *P. igniarius,* the content of this element was almost twice as high. The calcium content was also the lowest in *P. pini* and amounted to 128.02%, which is almost 2.5 times less than in the extract from *F. robusta*. The iron content in the extracts from *P. pini* and *P. igniarius* was at a similar level of about 5 mg/kg of extract weight. The iron content in the *F. robusta* extract was twice as high. The *F. robusta* extract contained the most zinc, while the *P. pini* extract contained the most copper. Interestingly, the *F. robusta* and *P. pini* extracts did not contain magnesium while, in the *Phellinus igniarius* extract, the content of this element was over 1 g/kg of extract weight. The highest silicon and sulfur content was noted in the *F. robusta* extract, and the lowest in *P. igniarius*. The tested extracts also contained arsenic of about 1–2 mg per kg of extracts. The presence of bromine and chlorine was also noted in the tested extracts. The latter element was observed in significant amounts in the *P. pini* and *P. igniarius* extracts—over 3 g/kg of extract weight—while in the *F. robusta* extract, the chlorine content was over 25 times lower. Small amounts of nickel were also observed in the extracts of *P. pini* and *P. igniarius*.

### 2.3. Thermal Analysis

**Figure 1:**
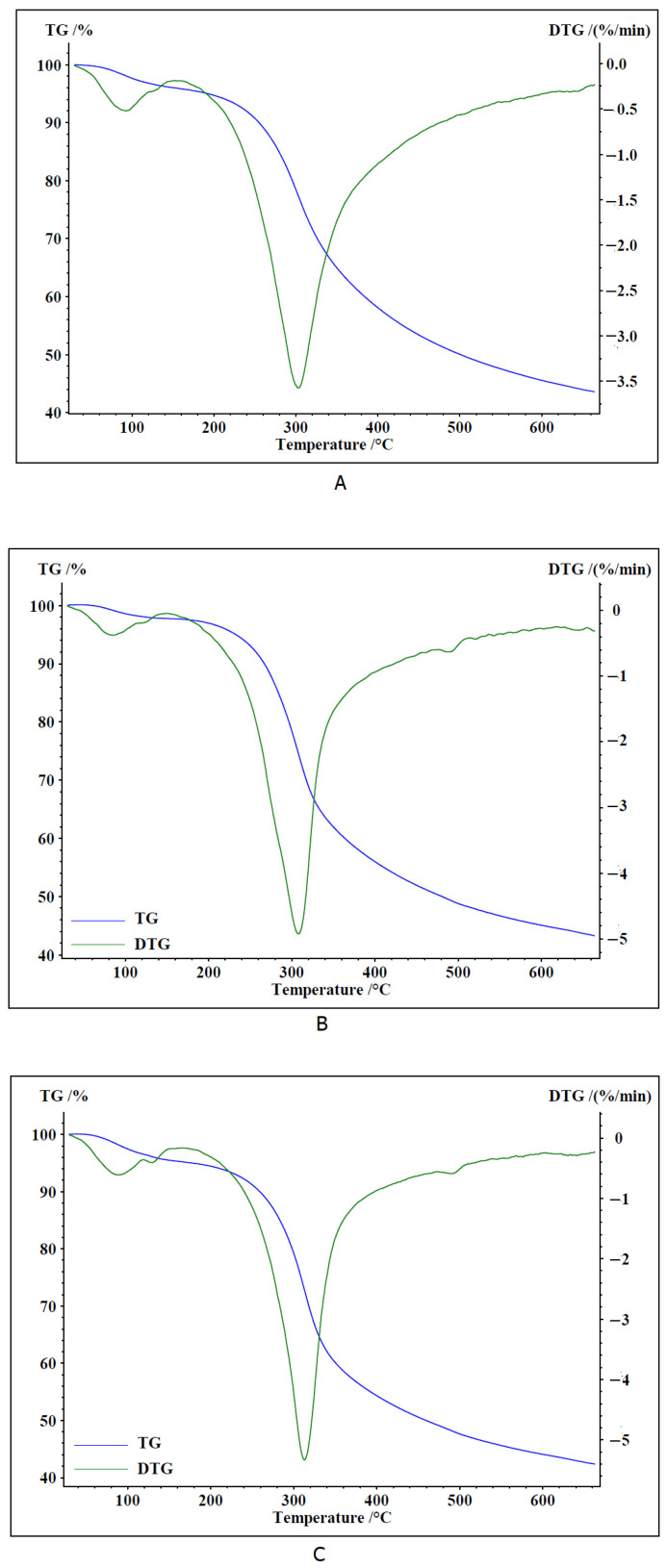
TG and DTG curves of dried fungal samples ((**A**)—*F. robusta*, (**B**)—*P. pini*, and (**C**)—*P. igniarius*).

Thermal analysis of dried fruiting bodies of *Fomitiporia robusta* (Figure 1A), *Porodaedalea pini* (Figure 1B), and *Phellinus igniarius* (Figure 1C) was performed. Dried and powdered fruiting bodies were heated in the temperature range of 30–660 °C in a nitrogen atmosphere. The first stage of thermal decomposition of the sample is evaporation of the remaining water. This process occurs in the temperature range of 50–200 °C. A rather mild decrease in mass is observed on the TG curves (Figure 1A–C) in this temperature range.

Based on the mass loss, it can be stated that the dried sample of *Fomitiporia robusta* contained about 4.1% water, *Porodaedalea pini* contained 2.2% water, and the water content in *Phellinus igniarius* was 4.8%. The peak on the DTG curve reached a minimum at 93.0 °C (*F. robusta*), 86.7 °C (*P. pini*), and 90.3 °C (*P. igniarius*).

Further heating of the samples led to the decomposition of organic substances contained in the samples—mainly sugars, carboxylic acids, and sterols. The maximum peak on the DTG decomposition curve of the *F. robusta* sample was 302.7.0 °C, while for the *P. pini* sample it was 307.9 °C, and for the *P. igniarius* sample it was 313.0 °C. The thermal decomposition process was carried out to a temperature of 660 °C. At a temperature of 640 °C, the thermal decomposition curves began to flatten out. The masses of the sample residues after thermal decomposition were 43.57% (Figure 1A), 43.22% (Figure 1B), and 42.30% (Figure 1C), respectively. The residue from the decomposition process of the tested dried fruiting body samples is the residue of inorganic carbon and mineral compounds contained in the tested samples. The thermal decomposition curves for all three species of the *Phellinus* genus have a similar course, which indicates a similar composition of the matter constituting the fruiting bodies of the tested fungi.

### 2.4. FTIR Spectroscopy

**Figure 2:**
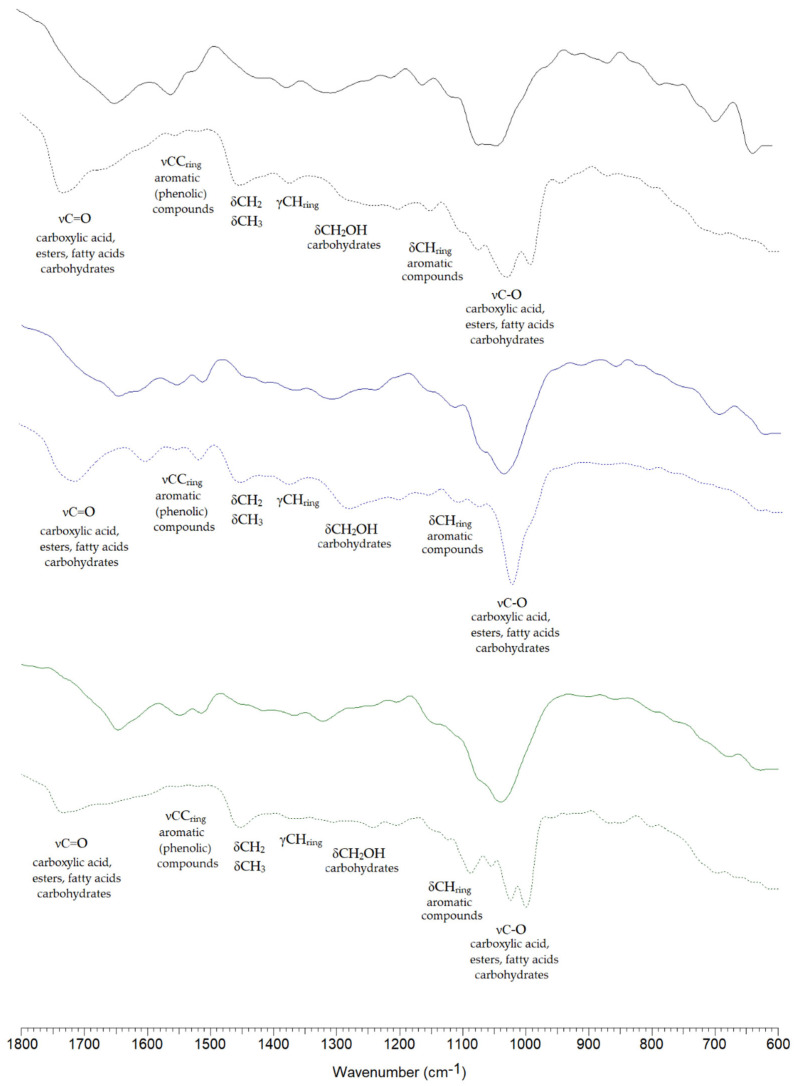
FTIR ATR spectra of *F. robusta* (black line), *P. pini* (blue line), and *P. igniarius* (green line). Continuous line—spectra of dried fruiting body; dashed line-spectra of methanolic extract.

Infrared spectroscopy (FTIR) was used to determine the main groups of organic compounds in the tested samples. Infrared spectra of dried fruiting bodies, *Phellinus igniarius*, *Fomitiporia robusta,* and *Porodaedalea pini,* and spectra of their extracts were recorded using the multireflection ATR technique in the spectral range of 2000–600 cm^−1^. The spectra are presented in Figure 2, while the position of the bands and their assignment are provided in Table 3.

In the FTIR spectra of the dried fruiting body samples and their extracts, characteristic bands of high intensity are present, which are a result of the stretching vibrations of the carbonyl group, νC=O. In the spectra of dried fruiting bodies, these bands are present in the spectral range of 1647–1645 cm^−1^, while in the spectra of fungal extracts, the bands are present in the range of 1735–1675 cm^−1^. The shift of these bands in the spectra of extracts is caused by the presence of carboxylic acid dimers in the dried fruiting body samples. Other characteristic bands present in the spectra are related to the stretching vibrations, νC-O. These bands are located in the wavenumber range of 1055–1024 cm^−1^. The presence of the νC=O and νC-O bands indicates the presence of compounds from the carboxylic acid group in the tested fruiting bodies and their extracts. Additionally, the spectra show bands associated with the vibrations of C-H and C-C bonds in aliphatic and aromatic systems. This indicates that the tested samples contain aromatic carboxylic acids and aliphatic carboxylic acids, including fatty acids.

Carbonylic group (νC-O) bands may also indicate the presence of carbohydrates, especially since the recorded spectra additionally show vibration bands originating from CH_2_OH groups. These include the δCH_2_OH bending vibration bands located in the wavenumber range of 1301–1242 cm^−1^. Other bands present in the spectra of dried fruiting bodies and extracts associated with the presence of carbohydrates are the δCOH, δCCH, and δOCH bending vibration bands located at wavenumbers 946–779 cm^−1^. In the spectra of the tested products, many bands originating from vibrations of the aromatic system—related to the presence of aromatic compounds—are also observed. These include bands originating from deformation vibrations δCHring in the wavenumber range of 1168–1088 cm^−1^, bands related to stretching vibrations νCC (1556–1514 cm^−1^), as well as bands related to deformation of the aromatic ring or de-fringing (697–624 cm^−1^).

### 2.5. GC/MS Analysis

**Figure 3:**
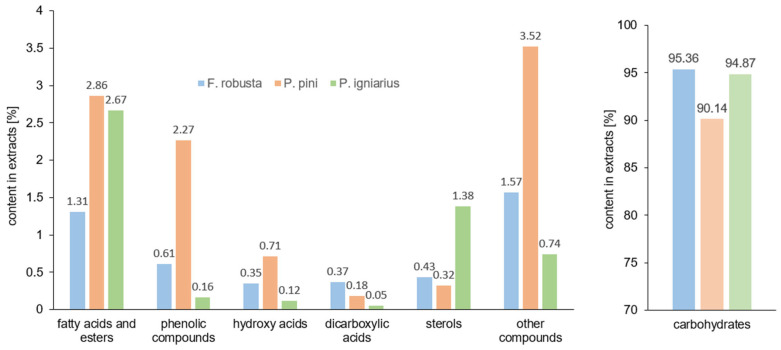
Main groups of compounds in the tested extracts of *F. robusta*, *P. pini*, and *P. igniarius*.

The results of GC/MS analysis showed that the main components of the extracts of *Phellinus igniarius*, *Fomitiporia robusta,* and *Porodaedalea pini* are carbohydrates. The contents of monosaccharides and disaccharides constituting the polysaccharide fraction are 95.36% (*F. robusta*), 94.87% (*P. igniarus*), and 90.14% (*P. pini*) (Figure 3). The structural characteristics of polysaccharides from samples of *Phellinus* spp. are generally defined by their average molecular weight, monosaccharide composition, and chemical structure [40,41]. The main disaccharide building block of the polysaccharides of the tested samples is trehalose, the content of which is about 58% in *F. robusta* and *P. pini*, and as much as 68% in *P. igniarius*. High contents of polyhydric alcohols were also noted, i.e., the content of mannitol was about 19% in *F. robusta* and *P. pini* and 13% in *P. igniarius*; the content of ribitol was almost 15% in the extract from *F. robusta*, 11% in *P. igniarius,* and 4% in *P. pini*. In the extract of *P. igniarius*, small amounts of sugars such as arabinitol, β-fructofuranose, α-glucopyranose, β-glucopyranose, myo-inositol, xylitol, and galactitol (with a total content of less than 1%) were also noted. In the extract of *F. robusta*, sugars such as arabinitol, α-glucopyranose, β-glucopyranose, myo-inositol, deoxy-inositol, galactitol, and sucrose were determined. In the extract of *P. pini*, the presence of the same sugars as in the extract of *F. robusta* was noted, as well as additional sugars such as pinitol (1.83%), threitol (0.74%), D-ribose (0.23%), fructose (0.23%), β-L-fucopyranose, and smaller amounts of β-mannopyranose and α-galactopyranose.

The content of fatty acids and their esters in the extracts of *Phellinus igniarius*, *Fomitiporia robusta,* and *Porodaedalea pini* was 2.67%, 1.31%, and 2.86%, respectively. The main acids identified in the extracts tested were palmitic acid (0.03%, 0.10%, and 0.59%, respectively), linoleic acid (0.20%, 0.35%, and 0.89%), and esters methyl palmitate (0.24%, 0.16%, and 0.25%) and methyl linoleate (1.07%, 0.47%, and 0.26%). In the extracts of *Phellinus igniarius* and *Porodaedalea pini*, the content of oleic acid (0.11% and 0.16%) was determined.

The highest content of phenolic acids was recorded in the extract from *P. pini* (2.27%), including 3-hydroxybenzoic acid (0.77%), protocatechuic acid (0.94%), caffeic acid (0.30%), and vanillic acid (0.10%), as well as small amounts of protocatechuic acid esters. These compounds are characterized by high antioxidant potential [42]. The *F. robusta* extract contained 0.61% of phenolic compounds, including 0.37% of 3-hydroxybenzoic acid, 0.12% of protocatechuic acid, and 0.05% of syringic acid. The lowest content of phenolic compounds was recorded in the extract from *P. igniarius* (0.16%), including 0.08% of 3-hydroxybenzoic acid, 0.05% of protocatechuic acid, and 0.04% of caffeic acid.

The extracts tested also contained compounds from the group of aliphatic hydroxy acids. The *P. igniarius* extract contained 0.12% of compounds from this group (lactic acid and malic acid), the *F. robusta* extract contained 0.35% (lactic acid, glycolic acid, malic acid, and citric acid), while the *P. pini* extract contained 0.71% (lactic acid, glycolic acid, glyceric acid, and malic acid).

The dicarboxylic acids present in the extracts tested were oxalic acid (only in *F. robusta*); succinic acid, which was present in all extracts in amounts below 0.15%; and fumaric acid, which was present in the amount of 0.27% in the *F. robusta* extract and 0.03% in the *P. igniarius* extract.

The sterol content in the *P. igniarius* extract was 1.38% (including ergosterol—0.9%, 3-hydroxyergosta-7,22-diene—0.27%, and 3-hydroxyergost-7-ene—0.12%). In the *F. robusta* extract, 0.43% sterols were noted (including 0.2% ergosterol, 0.14% 3-hydroxyergosta-7,22-diene, and 0.1% 3-hydroxyergost-7-ene). The *P. pini* extract contained 0.2% ergosterol and 0.11% 3-hydroxyergost-7-ene.

The extracts tested also contained small amounts of ethylene glycol (below 0.2%), 1,2-propanediol (below 0.15%), and pyroglutamic acid (below 0.2%). The glycerol content in *P. igniarius* was 0.51%, in *F. robusta* 0.19%, and in the extract from *P. pini* 1.42%. The extract from *P. pini* contained N-acetylglucosamine (0.59%), which is a component of biopolymers that build fungal cell walls.

### 2.6. Antioxidant Study

**Figure 4:**
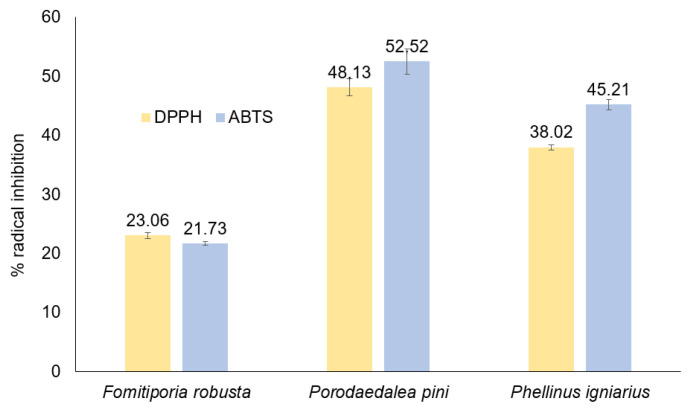
Antioxidant activity of *Phellinus* spp. extracts.

The antioxidant activity of methanolic extracts of *Fomitiporia robusta*, *Porodoedalea pini,* and *Phellinus igniarius* determined through tests with DPPH and ABTS radicals is presented in Figure 4. The concentration of extracts for which the level of radical inhibition was measured was 54 mg/L. The highest antioxidant activity in tests with ABTS and DPPH radicals was demonstrated by the extract from *P. pini*. Over 50% level scavenging of the ABTS radical and 48% level scavenging of the DPPH radical were noted. This extract was characterized by the highest content of phenolic compounds, which are mainly responsible for antioxidant activity. The lowest content of phenolic compounds was noted in the extract of *P. igniarius*; however, this extract did not show the lowest antioxidant potential among the extracts tested. A high content of sterols was found in this extract, which may also demonstrate antioxidant potential [43,44]. The level of ABTS radical neutralization by the *P. igniarius* extract was 45%, while that of the DPPH radical was 38%. The lowest antioxidant activity was demonstrated by the *F. robusta* extract, with 23% and 21.7% of radical removal, respectively, in the DPPH and ABTS tests. This extract is characterized by a low content of phenolic compounds, as well as a low content of sterols. For comparison, gallic acid at a concentration of 0.85 mg/L removes almost 100% of radicals in the ABTS test, and about 50% of radicals in the DPPH test.

**Figure 5:**
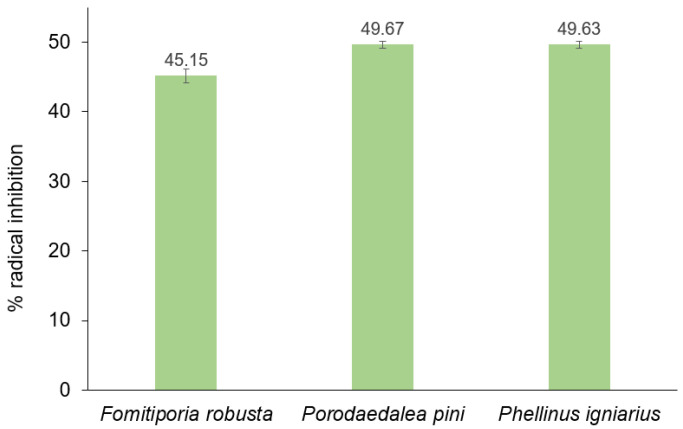
Lipid peroxidation inhibition after 5 days of reaction by *Phellinus* sample extracts.

Figure 5 presents the results of the fat oxidation inhibition test. The tested extracts at a concentration of 54 mg/L of *P. pini* and *P. igniarius* extracts caused nearly 50% inhibition of fat oxidation in the 5-day test. The *F. robusta* extract showed a slightly lower inhibition of oxidation fats (approximately 45%). The high sterol content in the *Phellinus igniarius* extract has a major impact on its antioxidant properties against fats.

### 2.7. Antimicrobial Study

**Figure 6:**
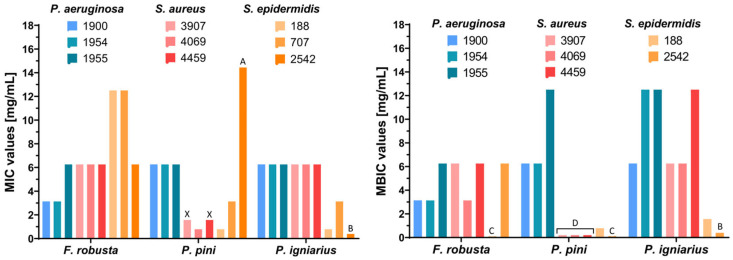
Antibacterial properties (MIC and MBIC) of *Fomitiporia robusta*, *Porodoedalea pini*, and *Phellinus igniarius* methanolic extracts against clinical bacterial strains; A—MIC > 12.5 mg/mL; B—MIC < 0.78 mg/mL; C—no inhibition of biofilm formation; D—MIC < 0.20 mg/mL; X—only for *S. aureus* 3907 and 4459 strains the MBC value was obtained (6.25 mg/mL).

To evaluate the therapeutic potential of tested fungal extracts, antimicrobial activity was assessed by determining the minimum inhibitory concentration (MIC), minimum bactericidal concentration (MBC), and minimum biofilm inhibitory concentration (MBIC). The MIC—the lowest concentration inhibiting bacterial growth—and the MBC—the concentration achieving bactericidal effects—were determined as described previously [45]. The MBIC is a concentration of tested agents that inhibits the formation of bacterial biofilm, which creates a protective barrier for bacteria, rendering them significantly more resistant to antibiotics [46]. Fungal extracts that inhibit biofilm formation can restore bacterial susceptibility to antibiotics, enhancing treatment efficacy. *Porodoedalea pini* (against *S. aureus* and *S. epidermidis*) and *P. igniarius* (against *S. epidermidis*) showed the highest anti-biofilm properties with MBIC values between 0.78 and 1.56 mg/mL against *Staphylococcus epidermidis* and <0.20 mg/mL against *S. aureus* clinical strains. *S. aureus* 4459 and 3907 strains showed increased resistance to the tested antibiotic, ERT, with MIC ≥ 0.50 mg/mL. Interestingly, in contrast to the other extracts tested, only the *P. pini* extract exhibited bactericidal activity against these two bacterial strains, as evidenced by its minimum bactericidal concentration (MBC) of 6.25 mg/mL. Furthermore, this extract demonstrated significant antibacterial activity with a minimum inhibitory concentration (MIC) of 1.56 mg/mL and a minimum biofilm inhibitory concentration (MBIC) below 0.20 mg/mL (Table 4, Figure 6). Planktonic bacteria—which are individual, free-floating cells—are more susceptible to antibacterial agents than biofilm bacteria, which form complex communities encased in a self-produced extracellular polymeric substance matrix. This matrix makes biofilm bacteria significantly more resistant, even up to 1000-fold [47]. *Phellinus iginarius* extract showed the lowest activity in inhibition of *P. aeruginosa* and *S. aureus* biofilm formation, which can be seen by comparing the MBIC results for all tested extracts. The lowest concentrations that inhibited biofilm formation of *S. aureus* and *S. epidermidis* were observed after treatment with *P. pini* extract, while *F. robusta* extract was the most effective against strains of *P. aeruginosa* (Figure 6).

### 2.8. Cytotoxicity

**Figure 7:**
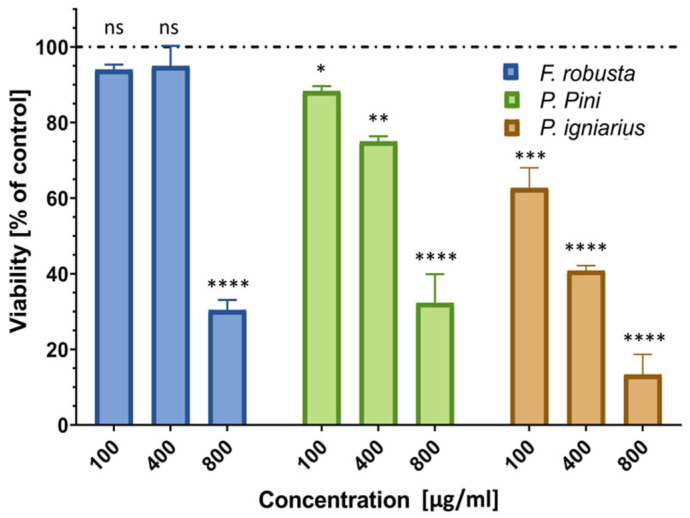
HaCaT cell viability after 24-h treatment with *Fomitiporia robusta*, *Porodoedalea pini*, and *Phellinus* igniarius methanolic extracts in the following concentrations: 100, 400, and 800 µg/mL. Viability was evaluated with the use of the NR assay. Statistical differences between the untreated control and cells treated with extracts were evaluated with the use of one-way ANOVA: * *p* < 0.05, ** *p* < 0.005, *** *p* < 0.002, and **** *p* < 0.0001.

The cytotoxicity of the examined fungal extracts was evaluated using the Neutral Red (NR) assay, which measures cell viability based on the uptake of NR dye by functional lysosomes [48]. Only *F. robusta* extract showed a lack of cytotoxicity against human keratinocytes at 100 and 400 µg/mL; however, the highest concentration (800 µg/mL) of this extract caused an approximately 70% reduction in cell viability. The other two extracts (*P. pini* and *P. igniarius*) showed varying cytotoxicity at all three concentrations, with the *P. igniarius* extract causing approximately 9, 43, and 69% decreases in HaCaT cell viability at concentrations of 100, 400, and 800, respectively (Figure 7).

### 2.9. Reactive Oxygen Species

**Figure 8:**
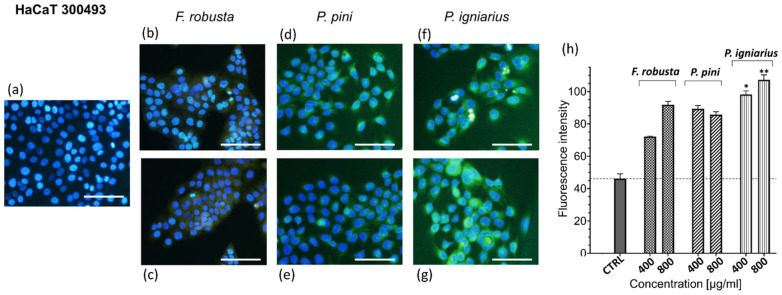
Fomitiporia robusta, Porodoedalea pini, and *Phellinus* igniarius extracts affect ROS production in HaCaT cells. Representative fluorescent images (40 ×) of CellROX ^®^Green Reagent-labeled cells, treated for 30 min: (**a**) untreated cells; 400 µg/mL (**b**) and 800 µg/mL (**c**) extracts of *F. robusta*; 400 µg/mL (**d**) and 800 µg/mL (**e**) extracts of *P. pini*; and 400 µg/mL (**f**) and 800 µg/mL (**g**) extracts of *Phellinus igniarius*; scale bar = 100 μm. Bar graph (**h**) summarizing the effect on ROS production after treatment with different extracts at concentrations of 400 and 800 µg/mL. Data are represented as mean ± SD; the presented results are not statistically significant, unless otherwise stated (* *p* < 0.05; ** *p* < 0.01).

Reactive oxygen species (ROS) generation is a critical mediator of cellular stress and oxidative damage that is implicated in diverse pathologies [49]. To investigate the effects of fungal extracts on intracellular ROS levels, the CellROX Green assay, a fluorogenic probe quantifying ROS through oxidation-induced fluorescence, was utilized. Only incubation with P. igniarius extract showed a statistically significant pro-oxidative effect against HaCaT cells (Figure 8).

## 3. Materials and Methods

### 3.1. Collection of Fungal Samples in the Field

This study investigated 30 samples belonging to 22 species of the genus *Phellinus sensu lato* (Table 5). Fruiting bodies were collected, among other specimens, in the Białowieża Forest (Poland); Village Kozhle (North Macedonia); Estremadura, Sesimbra, and Lagoa de Albufeira (Portugal); Zlatari close to Prishtina (Kosovo); and Spoleto and the Bosco Siro Negri State Nature Reserve (Italy). Each fruiting body found in the field was collected together with the substrate. Prior to collection, a photograph was taken under natural conditions, documenting the general habit and features of the fruiting body that may be lost during drying. The specimens were collected in paper envelopes on which the place and date of collection; the in situ fruiting body photo numbers; the type of substrate and host; and the habitat data were recorded. Additionally, in the case of saprobes growing on dead wood, the species was identified and information on the stage of decomposition of the substrate was recorded.

### 3.2. Morphological Identification

Slides were prepared under an OPTA-TECH MI6 binocular (OPTA-TECH) magnifier at 5–25x magnification. Thin sections of the fruiting body were obtained with a microtome or razor blade. If necessary, a preparation was made from the relevant part of the fruiting body—i.e., tube and context—and, for some species, also from the cap cover. The tube preparation was prepared in such a way as to make the following characteristics visible: the arrangement of the filaments in the trama; the appearance of the bristles in the hymenium and context, characteristic of the genus *Phellinus*; the appearance of the filament tips on the blades of the inner tube septa; and the appearance and elements of the hymenium at the bottom of the tubes. An OPTATECH LAB-40 light microscope with variable phase contrast and a NIKON ECLIPSE Ni with Nomarski contrast were used to observe microscopic features.

Preparations from fresh specimens were made in water and from dry specimens in 3–10% KOH. All available features were observed in these media. To improve observational comfort and obtain a more contrasting image, the slides were stained with Congo Red. This dye stains the cell walls and, less intensely, the cell contents. Given that microscopic features in the genus *Phellinus* s.l. have a dark brown color, lactophenol was used to lighten them. The amyloid and dextrorotatory properties of the microstructural elements were checked in Melzer’s reagent and in 0.1% Cotton Blue in 60% lactic acid to determine the degree of cyanophilicity of the spore walls and filaments.

The primary sources of information for the taxonomic analysis of fungi of the genus *Phellinus* s.l. were the following keys and monographic studies: Domanski et al. (1967, 1973) [50,51]; Ryvarden and Gilbertson (1993, 1994) [52,53]; Larsen and Cobb-Poulle (1990) [54]; and Sell (2008) [55]. The nomenclature was adopted from MycoBank(available online: http://www.mycobank.org (accessed on 26 March 2025)). The correctness of the spelling used and the author of the name at the genus level was confirmed with the latest edition of the *Dictionary of the Fungi* (Kirk et al. 2008) [56]. Acronyms of herbaria follow the Index Herbariorum. Documentary material (dry fungal specimens) was deposited in the herbarium of the Institute of Forest Sciences, Bialystok University of Technology (BLS).

### 3.3. Genetic Identification

In order to confirm genetic identity, DNA was isolated from the fungal samples with the Plant & Fungi DNA Purification Kit (EURx, Gdańsk, Poland). The obtained preparations were characterized spectrophotometrically (Varioskan LUX, Thermo Fisher Scientific, Waltham, MA, USA) and fluorometrically (Qubit 2.0, Thermo Fisher Scientific, US). Whole genome sequencing was performed using the DNA library prepared with the ITD xGen DNA EZ kit on an Illumina NovaSeq S4 system. Paired reads (2 × 150 bp) were cleared and then subjected to de novo assembly using MEGAHIT v. 1.2.9 and CLC Genomics Workbench v. 12.0.3. The resulting contigs were searched to identify reference sequences, including the small subunit ribosomal RNA gene fragment, the internal transcribed spacer 1, the 5.8 S ribosomal RNA gene, the internal transcribed spacer 2, and the partial large subunit ribosomal RNA gene used in taxonomic analyses. Sequences were deposited at NCBI (PV125346.1 and PV186754.1). The fungal material was dried.

### 3.4. Preparation of Extracts

The fruiting bodies were cleaned and separated from the remaining substrate and then dried at a temperature not exceeding 40 ˚C. The fruiting bodies were crushed in a laboratory grinder. The samples were then macerated in methanol (99.8%; POCH Avantor Performance Materials, Poland) at a ratio of 1:6 [g/mL]. The maceration period was no less than 2 months, and the samples were shaken systematically.

The finished tinctures were filtered through 80 g/m^2^ filter paper, and the solvent was evaporated in two steps. In the first step, most of the solvent was removed in a rotary evaporator Rotavapor^®^ R-100 (Büchi, Switzerland) at 46 °C, rotation speed 3–4 units, and under the reduced pressure from 300 to 100 mbar. In the second step, the extract was collected from the walls of the round-bottomed extraction flask together with a small amount of methanol and placed in the glass extraction cells of a parallel evaporation system, MultivaporTM P-12 (Büchi), where it was incubated at 46 °C and under reduced pressure from 400 to 100 mbar. Each solid-state extract was stored in the dark at 10 °C in the Fungi Extract Bank^®^ collection (available online: https://fungiextractbank.com/en (accessed on 1 April 2025)).

For fungal samples where the residue in the flask did not dissolve in methanol, the flask was rinsed with distilled water, then frozen at −25 °C, and lyophilized at −40 °C at a pressure of 0.04 mbar; these samples were stored for other tests. The methanolic extracts obtained had a plastic consistency, high viscosity, and colors from brown to dark brown. Dry methanolic extracts were used for further studies.

### 3.5. Antimicrobial Activity—Preliminary Studies

Preliminary screening tests of the antimicrobial activity of 30 samples of fungi were performed. Antimicrobial assays of fungal extracts were conducted as part of the project. For each extract, the MIC (minimum concentration of a substance that inhibits microbial growth) was determined using the microdilution method in Mueller–Hinton Broth II (MH II) medium using serial two-fold dilutions of the fungal extract, according to the procedure described by Balouiri et al. and Rašeta et al. [57,58]. The MIC value was expressed as the lowest concentration of fungal extract (in mg/mL) that completely inhibited the growth of the microorganism, as identified based on the turbidity of the culture after 24 h. To confirm the MIC value, cultures were performed on plates of three dilutions that showed the lowest or no turbidity. This study was performed on bacterial strains *Klebsiella pneumoniae* (ATCC-13883)**,** *Pseudomonas aeruginosa* (ATCC-27853)*, Staphylococcus aureus (*ATCC-25923)*, Staphylococcus epidermidis,* and *Kocuria rhizophila* (ATCC-9341), and fungi *Candida albicans (*ATCC-1023), which were obtained from the ATCC collection. Based on the MIC results, three extracts of *Phellinus* species were selected for further studies: *Fomitiporia robusta*, *Phellinus igniarius,* and *Porodaedalea pini*.

### 3.6. Elemental Analysis

**Figure 9:**
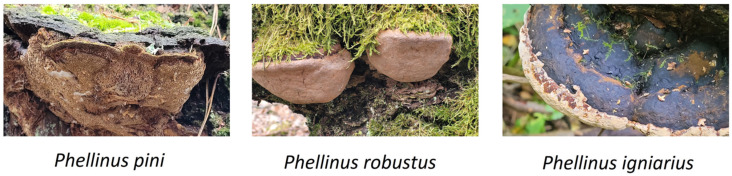
Fruiting bodies: *Phellinus pini* syn., *Porodaedalea pini*., *Phellinus robustus* syn. *Fomitiporia robusta*, and *Phellinus igniarius* (Photography: *M. Wołkowycki* and *M. Karadelev*).

The determination of selected elements in fungal extracts (*Phellinus igniarius, Fomitiporia robusta,* and *Porodaedalea pini* (Figure 9)) was performed using X-ray spectrofluorimetry. An amount of 900 µL of the aqueous extract solution was collected in an Eppendorf tube, and 100 µL of internal standard (Ga at 10 mg/L) was added to obtain a final concentration of 1 mg/L. An amount of 10 µL of the prepared solution was applied to measuring slides and dried at 50 °C. The prepared samples were analyzed using a TXRF (Total X-Ray Reflection Fluorescence) S2 PICOFOX apparatus, Bruker, Berlin, Germany.

### 3.7. Thermal Analysis

Thermal analysis of dried fruiting body samples of *Phellinus igniarius*, *Fomitiporia robusta,* and *Porodaedalea pini* was performed on a Libra thermal analyzer by TG 209 F1 (Netzsch company, Selb, Germany). Dried material weighing 10 ± 0.1 g was heated in ceramic crucibles in a nitrogen atmosphere (15 mL/min) with a temperature increase of 10 °C/min. The thermal decomposition process was carried out in the temperature range from 30 to 660 °C. The TG and DTG curves were recorded.

### 3.8. Gas Chromatography–Mass Spectrometry (GC-MS) Analysis

The fungus extract chemical composition was analyzed using an Agilent 7890A gas chromatograph coupled with an Agilent 5975C mass spectrometer. The methanol extract from fungus (10 mg) was diluted in 1 mL of pyridine, and 0.1 mL N,O-bis *(*trimethylsilyl*)-*trifluoroacetamide (BSTFA) was added. The sample was heated for 30 min at 60 °C. After silylation, 1 μL of the sample was entered into a GC/MS injector. The injector was set to a 1:10 split, and the temperature was 300 °C. The separation of compounds was conducted on a silica column HP-5MS (30 m × 0.25 mm × 0.25 μm), and the rate of helium flow was 1 mL/min. The starting column temperature was 50 °C, increased to 325 °C at 3 °C/min, and the final temperature was held for 10 min. The quadrupole and ion source temperatures were 150 °C and 230 °C, respectively. The energy of ionization was 70 eV, and detection was performed in a range of 41–800 units in a full scan mode. After peak integration, the percentage of every compound in the total ion current (% TIC) was calculated. Both mass spectra and retention indices were used for the identification of chemical compounds. The Isidorov (2020) [59], NIST (2020) [60], and Wiley (2020) [61] libraries of mass spectra were used for analysis. The experimental retention indices (RI_exp_.) were calculated in relation to the retention times of C8-C40 n-alkanes. The RI_exp_. values were compared with the literature retention indices (RI_lit._).

### 3.9. Spectroscopy

The FTIR spectra of both extracts and dried fruiting bodies of 3 selected fungal samples, *Phellinus igniarius, Fomitiporia robusta,* and *Porodaedalea pini,* were obtained using the ATR multireflection technique. The spectra were recorded in the range of 2000–600 cm^−1^ using an Alfa spectrometer (Bruker, Billerica, MA, USA).

### 3.10. Antioxidant Properties and Lipid Peroxidation Analysis

The antioxidant properties were determined using DPPH and ABTS radical assays.

*DPPH radical test*

The determination of antioxidant activity via the DPPH radical assay was conducted following the method described in [62]. Briefly, 1 mL of the extract was mixed with 2 mL of a DPPH radical solution. A control sample was prepared simultaneously, in which 1 mL of solvent (methanol) was used instead of the extract. The samples were incubated for 1 h. After incubation, absorbance was measured at 516 nm using a UV–VIS spectrophotometer (NANOCOLOR^®^ VIS, MACHEREY-NAGEL). Each sample was analyzed in triplicate. The free radical scavenging activity of DPPH was calculated based on absorbance measurements, with the average percentage of inhibition determined using the following equation:

(1) $$\%I=\frac{A_{c}^{516}-A_{s}^{516}}{A_{c}^{516}}\cdot 100\%$$

*%I*—percentage of inhibition; *A_c_*^516^—absorbance of the control sample at 516 nm; and *A_s_*^516^—absorbance of the sample containing the extract at 516 nm.

*ABTS radical test*

The antioxidant activity via the ABTS radical assay was determined based on the study by the authors of [63]. ABTS was prepared at a concentration of 7 mM and mixed with 2.45 mM of potassium persulfate (K_2_S_2_O_8_) in a 1:1 ratio, followed by incubation in the dark at 20 °C for approximately 12 h to generate the ABTS^+^ radical. For the assay, 1.5 mL of the working ABTS^+^ solution was diluted with 30 mL of methanol. Then, 1 mL of the extract was mixed with 1 mL of the ABTS^+^ solution. A control sample was prepared simultaneously, in which the extract was replaced with methanol. The samples were incubated for 7 min at 20 °C. After incubation, absorbance was measured at 734 nm using a UV–VIS spectrophotometer. Each sample was analyzed in triplicate. The average percentage of inhibition of the ABTS^+^ cation radical was calculated using the following equation:

(2) $$\%I=\frac{A_{c}^{734}-A_{s}^{734}}{A_{c}^{734}}\cdot 100\%$$

*%I*—percentage of inhibition; *A_c_*^734^—absorbance of the control sample at 734 nm; *A_s_*^734^—absorbance of the sample containing the extract at 734 nm.

*Lipid peroxidation*

To evaluate the ability of extracts to inhibit lipid peroxidation, following the method described in [64], a linoleic acid emulsion was prepared by mixing 0.312 mL of linoleic acid, 0.256 mL of Tween 20, and 0.05 M of phosphate buffer in a 50 mL volumetric flask, with the final volume adjusted using the buffer. Then, 1 mL of each extract, prepared at a concentration of 45 mg/25 mL, was added to 1.5 mL of the emulsion. Each extract was tested in five replicates, and control samples were prepared in parallel by replacing the extract with 1 mL of methanol. The test tubes were sealed and incubated at 40 °C. Once the target temperature was reached, 0.1 mL of the reaction mixture was sampled from each tube and mixed with 4.7 mL of 75% ethanol and 0.05 mL of 30% ammonium thiocyanate. After a 3-min reaction period, 0.05 mL of 0.02 M FeCl_2_ solution in 3.5% HCl was added. Absorbance was measured at 500 nm using a UV–VIS spectrophotometer, with 75% ethanol used as a reference. Measurements were performed every 24 h for 5 days, with 0.1 mL volumes withdrawn from the same emulsions at each time point. The percentage inhibition of linoleic acid peroxidation (%I) was calculated using the following formula:

(3) $$\%I=\frac{A_{c}^{500}-A_{s}^{500}}{A_{c}^{500}}\cdot 100\%$$

*%I*—percentage of peroxidation inhibition; *A_c_*^500^—absorbance of the control sample at 500 nm; and *A_s_*^500^—absorbance of the sample containing the extract at 500 nm.

### 3.11. Antibacterial Activity

*Bacterial Strains and Culture Conditions*

Clinical bacterial isolates, specifically *Pseudomonas aeruginosa* (1900, 1954, and 1955), *Staphylococcus aureus* (3907, 4069, and 4459), and *Staphylococcus epidermidis* (188, 707, and 2542), were obtained from the Department of Medical Laboratory Diagnostics, Provincial Specialist Hospital in Rzeszow, and deposited at the Department of Biotechnology and Bioinformatics, Faculty of Chemistry, Rzeszow University of Technology. All bacterial strains were cultured at 37 °C in a New Brunswick Innova 40 Shaker (Eppendorf AG, Hamburg, Germany) until reaching a turbidity equivalent to 0.5 McFarland standard (approximately 10^8^ colony-forming units per mL [CFU/mL]). The obtained cultures were then diluted to a final concentration of 10^5^ CFU/mL. All bacterial cultures were prepared under aseptic conditions within an ESCO Airstream Laminar Flow Cabinet.

*Antimicrobial Activity Assays*

Minimum inhibitory concentrations (MICs) were determined using a micro-broth dilution method in Mueller–Hinton Broth (MHB), as previously described [65]. Serial two-fold dilutions of extracts (0.39–25 mg/mL) were prepared in MHB. Following 24-h incubation at 37 °C, the MIC was defined as the lowest concentration inhibiting visible growth, confirmed by OD_600_ measurement (BIO-RAD microplate reader). Minimum bactericidal concentrations (MBCs) were determined by subculturing MIC-, 2xMIC-, and 4xMIC-treated cultures onto Mueller–Hinton Agar (MHA) for 24 h at 37 °C. The MBC was defined as a ≥ 99.9% reduction in CFU compared with the untreated controls. Erythromycin (ERT) susceptibility was assessed using the microdilution method (0.03–500 µg/mL).

*Anti-Biofilm Activity*

The ability of extracts to inhibit biofilm formation was assessed using the MTT assay. Bacterial cultures, conditions, and density of the culture, as well as the 96-well plate preparation with extract dilutions, were the same as for the MIC analysis (see above in Section 2.5 Antimicrobial Activity—Preliminary Studies). After 24-h incubation, non-adherent cells were removed, and adherent cells were stained with 0.5% MTT in PBS for 2 h. If bacteria in biofilm are alive and metabolically active, their oxidoreductase enzymes convert the yellow MTT dye into insoluble purple formazan crystals. These crystals accumulate within the cells or on the biofilm surface. To quantify the formazan, a solubilization solution (DMSO) was added to dissolve the crystals, forming a homogeneous purple solution [65]. Subsequently, the absorbance was measured at 600 nm (Varioskan™ LUX multimode microplate reader, Thermo Scientific, Waltham, MA, USA). The minimum biofilm inhibitory concentration (MBIC) was defined as the lowest concentration inhibiting biofilm formation compared with media-only controls. The experiments were conducted in triplicate.

### 3.12. Cytotoxic Study

*Cytotoxicity Assessment*

Human immortalized epidermal cells (HaCaT 300493, Cytion, Eppelheim, Germany) were cultured in DMEM supplemented with 10% FBS and 1% penicillin–streptomycin, at 37 °C with 5% CO_2_. Cell viability was determined via trypan blue exclusion using an Automatic Cell Counter TC20™ (Hercules, CA, USA) (Cytotoxicity was evaluated using the Neutral Red (NR) assay [48]. The cells were seeded in 96-well plates and exposed to extracts (100, 400, and 800 µg/mL in DMSO, final DMSO ≤1%) for 24 h. NR solution (0.033%) was added for 2 h; afterwards, plates were washed with PBS, and then destaining solution (50% ethanol, 49% water, and 1% acetic acid) was added. Absorbance was measured at 540 nm (Varioskan™ LUX multimode microplate reader, ThermoFisher Scientific, Waltham, MA, USA). Cell viability was expressed as a percentage of the untreated controls.

*Reactive Oxygen Species (ROS) Quantification*

HaCaT cells were treated with *Fomitiporia robusta*, *Porodaedalea pini*, and *Phellinus igniarius* extracts (400 and 800 µg/mL) for 2 h. Non-treated cells were used as a negative control. ROS production was assessed using the CellROX™ Green Oxidative Stress kit (ThermoFisher Scientific, Waltham, MA, USA). The cells were incubated with 5 µM CellROX Green for 30 min, fixed with 4% paraformaldehyde, and counterstained with Hoechst 33342. Fluorescence imaging was performed using the cellSens Software 4.3 (Olympus, Center Valley, PA, USA). CellROX fluorescence intensity was quantified using ImageJ 1.54 g [66].

### 3.13. Statistical Analysis

The final results obtained from triplicate experiments are presented as means ± SD. GraphPad Prism version 8.0.1 (GraphPad Software, Boston, MA, USA, available online: http://www.graphpad.com (accessed on 20 April 2025)) was used to compare the differences between groups. Statistical significance was indicated for *p*-values < 0.05.

## 4. Conclusions

Antimicrobial activity studies of 30 samples of methanol extracts (22 *Phellinus* species) of fungi from the genus *Phellinus* allowed for the identification of extracts with the highest activity. The identified extracts were subjected to further detailed studies. The antimicrobial activity of fungi from the *Porodaedalea pini*, *Fomitiporia robusta*, and *Phellinus igniarius* species was tested on strains of multidrug-resistant bacteria. It was observed that the habitat and origin of a given species have a significant influence on biological activity (related to the content of biologically active compounds). Studies of the content of active compounds in the analyzed extracts showed that each of the three selected extracts was characterized by a high content of sugars (over 90% of the extract mass). *Porodaedalea pini* extract was characterized by the highest content of phenolic compounds among the three samples analyzed in detail, which determined the highest antioxidant activity of the extract of this mushroom in antioxidant tests.

Antimicrobial tests performed on antibiotic-resistant bacteria (*Pseudomonas aeruginosa*, *Staphylococcus aureus,* and *Staphylococcus epidermidis*) showed that *P. pini* extract was characterized by the highest activity in neutralizing these microorganisms among the three extracts tested. Cytotoxicity tests of the three selected extracts from fungi of the *Phellinus* species against human keranocytes showed that only the *F. robusta* extract did not show a cytotoxic effect, while the remaining two extracts (*P. pini* and *P. igniarius*) were biocidal against keranocyte cell lines.

The effects of the three mushroom extracts on the level of reactive oxygen species (ROS) generation—which are critical mediators of cellular stress and oxidative damage associated with various pathologies—were also studied. The studies were performed on the human keratinocyte HaCaT cell line. It was observed that only the *P. igniarius* extract showed a statistically significant pro-oxidant effect against HaCaT cells.

The conducted experiments provided promising results that are expected to form the basis of further studies on the biological activity of *Phellinus sensu lato* fungi. It is anticipated that the use of other methods for extracting biologically active compounds from *Phellinus* fungi will allow for obtaining extracts with high biological activity, which may result in the application of these extracts as compounds with high antimicrobial, antioxidant, and/or anticancer potential.

## Figures and Tables

**Table 1:** Antimicrobial properties (MIC) of examined *Phellinus* spp. methanolic extracts.

Sample No.	Species Name	*C. * *albicans* [mg/mL]	*P. * *aeruginosa* [mg/mL]	*S. * *aureus* [mg/mL]	*S. * *epidermidis* [mg/mL]	*K. * *pneumoniae* [mg/mL]	*K. * *rhizophila* [mg/mL]
16.D	*Porodaedalea chrysoloma*	>50	>50	50	>50	50	>50
17.D	*Porodaedalea pini*	>50	50	25	50	50	50
18.D	*Phellinus tremulae*	>50	50	12,5	50	50	50
19.D	*Phellinus laevigatus*	>50	>50	50	>50	>50	>50
21.D	*Phellinus tuberculosus*	>50	>50	25	>50	50	>50
22.D	*Fomitiporia robusta*	>50	50	25	25	50	25
25.D	*Phellinopsis conchata*	25	>50	25	>50	50	50
38.D	*Phellinus igniarius*	>50	>50	25	>50	>50	50
118.D	*Phellinus alni*	25	>50	25	>50	>50	50
160.D	*Fuscoporia ferruginosa*	50	50	25	50	50	25
254.D	*Phellinus alni*	25	>50	25	>50	>50	>50
255.D	*Phellinus alni*	50	>50	>50	50	>50	50
256.D	*Phellinidium ferrugineofuscum*	25	>50	50	>50	>50	50
257.D	*Fomitiporia punctata*	>50	50	50	50	50	50
258.D	*Fomitiporia punctata*	>50	50	>50	50	25	25
261.D	*Phellinus nigricans*	>50	>50	25	50	>50	>50
262.D	*Phellinus populicola*	>50	50	12.5	>50	50	50
266.D	*Fomitiporia robusta*	>50	>50	25	50	50	25
267.D	*Phellinus nigricans*	>50	>50	12.5	50	50	50
268.D	*Fomitiporia hartigii*	>50	50	25	25	50	25
279.D	*Phellinus tuberculosus*	50	>50	25	>50	>50	>50
280.D	*Fomitiporia robusta*	25	50	25	50	50	25
298.D	*Porodaedalea pini*	12.5	50	25	25	50	25
300.D	*Phellinus nigricans*	25	>50	25	50	>50	>50
300.Da	*Phellinus nigricans*	>50	>50	6.3	50	>50	50
301.D	*Phellinus igniarius*	25	>50	6.3	25	50	50
319.D	*Fuscoporia torulosa*	50	>50	50	50	50	>50
320.D	*Fuscoporia torulosa*	25	>50	>50	50	50	50
322.D	*Fomitiporia mediterranea*	>50	50	50	50	50	50
329.D	*Porodaedalea chrysoloma*	50	>50	12.5	50	50	50

**Table 2:** Elemental composition of examined *Phellinus* spp. extracts.

Element	Content of Elements [mg/kg of Extract]					
	*Fomitiporia robusta*	* SD	*Porodaedalea pini*	* SD	*Phellinus igniarius*	* SD
Cu	17.50	0.75	23.18	0.83	6.01	0.22
Fe	10.84	0.69	5.35	0.22	5.04	0.88
Mn	10.86	0.79	1.69	0.10	-	-
Ni	-	-	0.41	0.14	0.57	0.14
Zn	18.52	0.27	16.15	0.79	6.01	0.22
Ti	5.76	0.28	4.92	0.27	8.09	1.02
Rb	12.81	0.55	11.47	0.70	18.27	0.72
K	3218.03	0.50	2349.73	5.62	2287.27	2.91
Ca	324.89	1.44	128.02	2.91	146.06	1.70
Mg	-	-	-	-	1051.27	1.90
P	2930.88	1.87	1224.77	2.40	2117.81	0.94
S	710.19	0.64	581.22	0.94	252.63	1.13
Cl	139.01	0.35	3194.66	1.00	3240.80	0.62
Si	202.47	0.89	88.55	1.90	70.70	2.40
As	1.78	0.48	1.41	0.09	0.99	0.11
Br	3.80	0.23	21.54	2.22	4.18	0.38

* SD: standard deviation.

**Table 3:** Wavenumbers (cm^−1^), intensities, and assignments of bands occurring in the FTIR spectra of dried fruiting body samples and extracts of *F. robusta*, *P. pini*, and *P. igniarius*.

*Fomitiporia robusta*		*Porodaedalea pini*		*Phellinus igniarius*		Type of Vibration	Groups of Chemical Compounds
Dried Fruiting Body	Extract	Dried Fruiting Body	Extract	Dried Fruiting Body	Extract		
1645 s	1733 s	1647 s	1711 vs	1647 s	1735 s	νC = O	Carboxylic acid, esters, fatty acids, and carbohydrates
	1675 m						
1556 m	1554 m	1555 m	1552 m	1549 m	1553 w	νCC_ring_	Aromatic/phenolic compounds
1515 m	1520 m	1514 m	1517 m	1515 m	1519 w	νCC_ring_	Aromatic/phenolic compounds
-	1454 m	-	1451 s	-	1453 m	δCH_2_, δCH_3_	Aromatic and aliphatic compounds
-	-	1415 m	1410 m	1416 m	-	δCH_2_, δCH_3,_ δOH	Aromatic and aliphatic compounds, and carbohydrates
1372 m	1374 m	1365 m	1374 m	1366 m	1374 m	γCH_ring_	Aromatic/phenolic compounds
1301 m	-	1310 w	1280 m		1306 m	δCH_2_OH	Carbohydrates
		1242 w		1245 m	1243 m	δCH_2_OH	Carbohydrates
1206 w	1202 m	-	1202 m	1205 m	1205 m	νCO, νCC, δCOH	Carbohydrates
1168 m	1151 m	-	1155 m	-		δCH_ring_	Aromatic/phenolic compounds
	1113 m	1116 m	1107 m	-	1123 m	δCH_ring_	Aromatic/phenolic compounds
-	1075 m	-	1075 m	-	1088 s	δCH_ring_	Aromatic/phenolic compounds
1054 vs, 1037 vs	1029 vs	1038 s	1024 s	1041 s	1055 s	νC-O	Carboxylic acids, fatty acids, and carbohydrates
-	993 m	-	-	-	1000 s		
913 w	946 w	915 w	-	899 m	-	δCOH, δCCH, δOCH	Carbohydrates
862 m	871 m	860 m	874 m	858 m	865 m	δCOH, δCCH, δOCH	Carbohydrates
779 m	779 m	-	809 m	-	800 m	δCOH, δCCH, δOCH	Carbohydrates
691 m	691 s	697 s	-	-	695 m	De-fring	Aromatic/phenolic compounds
632 s	657 s	624 m	633 m	627 s	635 s m	De-fring	Aromatic/phenolic compounds

ν—stretching, δ—deforming in plane, γ—deforming out of plane, ring—vibrations of atoms of an aromatic ring, and intensities: vs—very strong, s—strong, m—medium, w—weak.

**Table 4:** Antibacterial properties (MIC, MBC, and MBIC) of *Fomitiporia robust*a, *Porodoedalea pini*, and *Phellinus igniarius* methanolic extracts against clinical bacterial strains.

Methanolic Extract	Concentration [mg/mL]	Clinical Strains								
		*Pseudomonas* *aeruginosa*			*Staphylococcus aureus*			*Staphylococcus* *epidermidis*		
		1900	1954	1955	3907	4069	4459	188	707	2542
*F. robusta*	MIC	3.13	3.13	6.25	6.25	6.25	6.25	>6.25	>6.25	6.25
	MBC	BS ^a^	BS ^a^	BS ^a^	BS ^a^	BS ^a^	BS ^a^	BS ^a^	BS ^a^	BS ^a^
	MBIC	3.13	3.13	6.25	6.25	3.13	6.25	- ^c^	- ^b^	6.25
*P*. *pini*	MIC	6.25	6.25	6.25	1.56	0.78	1.56	0.78	3.13	>12.5
	MBC	BS ^a^	BS ^a^	BS ^a^	6.25	BS ^a^	6.25	BS ^a^	BS ^a^	BS ^a^
	MBIC	6.25	6.25	12.50	<0.20	<0.20	<0.20	0.78	- ^b^	- ^c^
*P. igniarius*	MIC	6.25	6.25	6.25	6.25	6.25	6.25	0.78	3.13	<0.78
	MBC	BS ^a^	BS ^a^	BS ^a^	BS ^a^	BS ^a^	BS ^a^	BS ^a^	BS ^a^	BS ^a^
	MBIC	6.25	12.50	12.50	6.25	6.25	12.50	1.56	- ^b^	<0.78
ERT	MIC	0.06	0.06	0.06	0.50	2 × 10^−4^	>0.50	2 × 10^−4^	2 × 10^−3^	2 × 10^−4^
	MBC	BS ^a^	BS ^a^	BS ^a^	BS ^a^	BS ^a^	>0.50	4 × 10^−3^	2 × 10^−2^	4 × 10^−3^
	MBIC	0.06	0.06	0.06	0.50	2 × 10^−4^	- ^c^	2 × 10^−4^	- ^b^	2 × 10^−4^

^a^ Bacteriostatic (BS) properties of compounds for which the MIC value was determined. ^b^ This bacterial strain does not form biofilm. ^c^ No inhibition of biofilm formation.

**Table 5:** Characteristics of evidentiary specimens of *Phellinus* spp. samples.

Sample No.	Evidence Specimen Number	Species Name	Substrate/Host (Species Name)	Collection Site	Collected and Identified
16.D	BLS-M-05505	***Porodaedalea chrysoloma*** *(Fries) Fiasson & Niemelä [syn. Phellinus chrysoloma (Fr.) Donk]*	*Picea abies*	Poland, Białowieża Forest	Leg. et det. Marek Wołkowycki
17.D	BLS-M-05498	***Porodaedalea pini*** *(Brot.) Murrill [syn.* *Phellinus pini (Brot.) A. Ames]*	*Pinus sylvestris*	Poland, Białowieża Forest	Leg. et det. Marek Wołkowycki
18.D	BLS-M-05506	***Phellinus tremulae*** *(Bondartsev) Bondartsev & P.N. Borissov*	*Populus* *tremula*	Poland, Białowieża Forest	Leg. et det. Marek Wołkowycki
19.D	BLS-M-05512	***Phellinus laevigatus*** *(Fries) Bourdot & Galzin*	*Betula pendula*	Poland, Białowieża Forest	Leg. et det. Marek Wołkowycki
21.D	BLS-M-05493	***Phellinus tuberculosus*** *(Baumgarten) Niemelä [syn. Phellinus pomaceus (Pers.) Maire]*	*Prunus* *domestica*	Poland, Białowieża Forest	Leg. et det. Marek Wołkowycki
22.D	BLS-M-05497	***Fomitiporia robusta*** *(P. Karst.) Fiasson & Niemelä [syn. Phellinus robustus (P. Karst.) Bourdot & Galzin]*	*Quercus robur*	Poland, Białowieża Forest	Leg. et det. Marek Wołkowycki
25.D	BLS-M-02596	***Phellinopsis conchata*** *(Persoon) Dai [syn. Phellinus conchatus (Pers.: Fr.) Quél]*	*Salix caprea*	Poland, Białowieża Forest	Leg. et det. Marek Wołkowycki
38.D	BLS-M-01027	***Phellinus igniarius*** *(L.) Quél.*	*Salix fragilis*	Poland, Sokolskie Hill, Losiniany	Leg. et det. Marek Wołkowycki
118.D	BLS-M-05609	***Phellinus alni*** *(Bondartsev) Parmasto*	*Alnus glutinosa*	Poland, Białowieża Forest	Leg. et det. Marek Wołkowycki
160.D	BLS-M-05495	***Fuscoporia ferruginosa*** *(Schrad ex J.F. Gmelin) Murill [syn. Phellinus ferruginosus (Schrad.: Fr.) Pat.]*	*Fraxinus* *excelsior*	Poland, Białowieża Forest	Leg. et det. Marek Wołkowycki
254.D	BLS-M-05492	***Phellinus alni*** *(Bondartsev) Parmasto*	*Malus* *domestica*	Poland, Białowieża Forest	Leg. et det. Marek Wołkowycki
255.D	BLS-M-05490	***Phellinus alni*** *(Bondartsev) Parmasto*	*Carpinus* *betulus*	Poland, Białowieża Forest	Leg. et det. Marek Wołkowycki
256.D	BLS-M-05448	***Phellinidium ferrugineofuscum*** *(P. Karst) Fiasson & Niemelä [syn. Phellinus ferrugineofuscus (P. Karst.) Bourdot]*	*Picea abies*	Poland, Białowieża Forest	Leg. et det. Marek Wołkowycki
257.D	BLS-M-05494	***Fomitiporia punctata*** *(Fries ex P. Karsten) Murill [syn. Phellinus punctatus (P.Karst.) Pilát]*	*Corylus avellana*	Poland, Białowieża Forest	Leg. et det. Marek Wołkowycki
258.D	BLS-M-05499	***Fomitiporia punctata*** *(Fries ex P. Karsten) Murill [syn. Phellinus punctatus (P.Karst.) Pilát]*	*Sorbus* *aucuparia*	Poland, Białowieża Forest	Leg. et det. Marek Wołkowycki
261.D	BLS-M-05509	***Phellinus nigricans*** *(Fries) P. Karsten*	*Betula pendula*	Poland, Białowieża Forest	Leg. et det. Marek Wołkowycki
262.D	BLS-M-05507	***Phellinus populicola*** *Niemelä*	*Populus* *tremula*	Poland, Białowieża Forest	Leg. et det. Marek Wołkowycki
266.D	BLS-M-02977	***Fomitiporia robusta*** *(P. Karst.) Fiasson & Niemelä [syn. Phellinus robustus (P. Karst.) Bourdot & Galzin]*	*Robinia* *pseudoacacia*	Poland, Białowieża Forest	Leg. et det. Marek Wołkowycki
267.D	BLS-M-04016	***Phellinus nigricans*** *(Fries) P. Karsten*	*Betula* *pubescens*	Poland, Białowieża Forest	Leg. et det. Marek Wołkowycki
268.D	BLS-M-05496	***Fomitiporia hartigii*** *(Allesch. & Schnabl) Fiasson & Niemelä [syn. Phellinus hartigii (Allesch. & Schnabl) Pat]*	*Abies alba*	Poland, Beskid Niski	Leg. et det. Anna Hreczka rev. Marek Wołkowycki
279.D	BLS-M-05599	***Phellinus tuberculosus*** *(Baumgarten) Niemelä [syn. Phellinus pomaceus (Pers.) Maire]*	*Cerasus avium*	Poland, Kuraszewo, wild fruit orchard	Leg. Konrad Wilamowski det. Marek Wołkowycki
280.D	BLS-M-05610	***Fomitiporia robusta*** *(P. Karst.) Fiasson & Niemelä [syn. Phellinus robustus (P. Karst.) Bourdot & Galzin]*	*Quercus rubra*	Poland, Białowieża Forest	Leg. et det. Marek Wołkowycki
298.D	BLS-M-10167	***Porodaedalea pini*** *(Brot.) Murrill (syn. Porodaedalea pini (Brot.) A. Ames)*	*Pinus pinea*	Portugal, Estremadura, Sesimbra, Lagoa de Albufeira	Leg. et det. Mitko Karadelev
300.D	BLS-M-10094	***Phellinus nigricans*** *(Fries) P. Karsten*	*Fagus sylvatica*	Poland, Białowieża Forest	Leg. Konrad Wilamowski det. Marek Wołkowycki
300.Da	BLS-M-10164	***Phellinus nigricans*** *(Fries) P. Karsten*	*Fagus sylvatica*	Poland, Baltic Coast, “Zagorska Struga” Valley	Leg. et det. Mirosław Wantoch-Rekowski rev. Marek Wołkowycki
301.D	BLS-M-10093	***Phellinus igniarius*** *(L.) Quél.*	*Sorbus* *intermedia*	Poland, Romnicka Forest	Leg. Konrad Wilamowski det. Marek Wołkowycki
319.D	BLS-M-10412	***Fuscoporia torulosa*** *(Pers.) T. Wagner & M. Fisch [syn. Phellinus torulosus (Pers.) Bourdot & Galzin]*	*Quercus ilex*	Italy, Perugia, Sacro Bosco di Monteluco, Spoleto	Leg. et det. Carolina Girometta
320.D	BLS-M-10413	***Fuscoporia torulosa*** *(Pers.) T. Wagner & M. Fisch [syn. Phellinus torulosus (Pers.) Bourdot & Galzin]*	*Robinia* *pseudoacacia*	Italy, Pavia, “Bosco Siro Negri” State Nature Reserve	Leg. et det. Carolina Girometta
322.D	BLS-M-10414	***Fomitiporia mediterranea*** *M. Fisch. [syn. Phellinus punctatus (P. Karst.) Pilát]*	*Robinia* *pseudoacacia*	Italy, Pavia, “Bosco Siro Negri” State Nature Reserve	Leg. et det. Carolina Girometta
329.D	BLS-M-05505	***Porodaedalea chrysoloma*** *(Fr.) [syn. Porodaedalea abietis]* *[syn. Phellinus chrysoloma (Fr.) Donk]*	*Picea abies*	Poland, Białowieża Forest	Leg. et det. Marek Wołkowycki

## Data Availability

The data presented in this study are available upon request from the corresponding author.
